# Overexpression of GATA1 Confers Resistance to Chemotherapy in Acute Megakaryocytic Leukemia

**DOI:** 10.1371/journal.pone.0068601

**Published:** 2013-07-10

**Authors:** John Timothy Caldwell, Holly Edwards, Alan A. Dombkowski, Steven A. Buck, Larry H. Matherly, Yubin Ge, Jeffrey W. Taub

**Affiliations:** 1 MD/PhD Program, Wayne State University School of Medicine, Detroit, Michigan, United States of America; 2 Cancer Biology Program, Wayne State University School of Medicine, Detroit, Michigan, United States of America; 3 Department of Oncology, Wayne State University School of Medicine, Detroit, Michigan, United States of America; 4 Molecular Therapeutics Program, Barbara Ann Karmanos Cancer Institute, Wayne State University School of Medicine, Detroit, Michigan, United States of America; 5 Division of Pharmacology and Toxicology, Children’s Hospital of Michigan, Detroit, Michigan, United States of America; 6 Department of Pediatrics, Wayne State University School of Medicine, Detroit, Michigan, United States of America; 7 Division of Pediatric Hematology/Oncology, Children’s Hospital of Michigan, Detroit, Michigan, United States of America; 8 Department of Pharmacology, Wayne State University School of Medicine, Detroit, Michigan, United States of America; INRS, Canada

## Abstract

It has been previously shown that acute myeloid leukemia (AML) patients with higher levels of GATA1 expression have poorer outcomes. Furthermore, pediatric Down syndrome (DS) patients with acute megakaryocytic leukemia (AMKL), whose blast cells almost universally harbor somatic mutations in exon 2 of the transcription factor gene *GATA1*, demonstrate increased overall survival relative to non-DS pediatric patients, suggesting a potential role for GATA1 in chemotherapy response. In this study, we confirmed that amongst non-DS patients, GATA1 transcripts were significantly higher in AMKL blasts compared to blasts from other AML subgroups. Further, GATA1 transcript levels significantly correlated with transcript levels for the anti-apoptotic protein Bcl-xL in our patient cohort. ShRNA knockdown of GATA1 in the megakaryocytic cell line Meg-01 resulted in significantly increased cytarabine (ara-C) and daunorubicin anti-proliferative sensitivities and decreased Bcl-xL transcript and protein levels. Chromatin immunoprecipitation (ChIP) and reporter gene assays demonstrated that the *Bcl-x* gene (which transcribes the Bcl-xL transcripts) is a *bona fide* GATA1 target gene in AMKL cells. Treatment of the Meg-01 cells with the histone deacetylase inhibitor valproic acid resulted in down-regulation of both GATA1 and Bcl-xL and significantly enhanced ara-C sensitivity. Furthermore, additional GATA1 target genes were identified by oligonucleotide microarray and ChIP-on-Chip analyses. Our findings demonstrate a role for GATA1 in chemotherapy resistance in non-DS AMKL cells, and identified additional GATA1 target genes for future studies.

## Introduction

In the pediatric population, acute myeloid leukemia (AML) has a relatively guarded prognosis with five-year survival rates of approximately 50% (www.seer.cancer.gov), despite intensive therapy. Acute megakaryocytic leukemia (AMKL; M7) is a biologically heterogeneous form of AML, representing ~10% of pediatric AML cases and 1-2% of adult AML cases [1,2]. AMKL is considered a very high-risk subgroup with event-free survival (EFS) rates of <35% [3,4]. Remarkably, Down syndrome (DS) children with AML, and in particular, AMKL, have extremely high EFS rates of approximately 80% [3,5–8]. The blast cells of DS AMKL patients almost universally harbor a somatic mutation in exon 2 of the transcription factor *GATA1* gene (localized to Xp11.23), resulting in the introduction of premature stop codons and the synthesis of a shorter GATA1 protein (designated GATA1s, 40-kDa) initiated from a downstream initiation site and distinguishable from the wild-type GATA1 (50-kDa) [9]. Both GATA1s and the wild-type GATA1 proteins show similar DNA binding abilities and interact with a partner protein named “friend of GATA1” (FOG1), though the GATA1s protein exhibits altered transactivation capacity due to the loss of the N-terminal activation domain [9].

GATA1 is a zinc finger transcription factor that is essential for hematopoiesis of the erythrocyte/megakaryocyte lineages. GATA1 acts as an activator or repressor of different target genes by forming distinct activating or repressive complexes with its partner proteins (reviewed in 10). The pronounced differences in clinical outcomes between DS and non-DS AMKL patients and differences in the *GATA1* gene mutation status in blast cells suggest a potential role for GATA1 in chemotherapy response in both DS and non-DS AMKL cases. In the non-DS population, overexpression of GATA1 in megakaryoblasts from children with AMKL compared to blasts from children with other subtypes of AML was previously observed in gene expression microarray studies [11]. Further, earlier studies demonstrated a worse prognosis for AML patients (adults without AMKL) whose blast cells expressed higher levels of GATA1 than patients whose blasts expressed lower levels of GATA1 [12,13]. Collectively, these studies suggest that GATA1 may contribute to chemotherapy resistance via regulation of GATA1 target genes in AML, especially in the AMKL subtype.

Bcl-xL, encoded by the long form splice variant of *Bcl-x* transcripts which counteracts apoptotic signals, may be one of these GATA1 target genes. Bcl-xL is a Bcl-2 family protein that is abundantly expressed in both megakaryocytes and erythrocytes (reviewed in 14). Bcl-xL deficient mice exhibit massive apoptosis of fetal liver hematopoietic cells, suggesting that Bcl-xL prevents apoptosis of hematopoietic cells [15]. Previous studies have established that GATA1 and erythropoietin cooperate to promote erythroid cell survival by regulating Bcl-xL expression [16], and that GATA1 is capable of binding and activating the Bcl-xL promoter during erythroid differentiation [17]. Thus, it is conceivable that GATA1 may also regulate Bcl-xL in megakaryocytes as megakaryocytes and erythrocytes are derived from a common progenitor and both Bcl-xL and GATA1 are expressed in megakaryocytes.

In this study, we confirmed the overexpression of *GATA1* transcripts in non-DS megakaryoblasts compared to non-DS AML blasts. We also demonstrated that GATA1 plays critical roles in sensitivities of megakaryocytic cells to cytarabine (ara-C) and daunorubicin (DNR), the two main drugs used for treating AML, through direct regulation of Bcl-xL. Furthermore, we found that the histone deacetylase (HDAC) inhibitor, valproic acid (VPA), can decrease GATA1 expression and synergize with ara-C in exerting antileukemic activities toward megakaryocytic leukemia cells. Using gene-expression microarray and ChIP-on-Chip analyses, we identified additional GATA1 target genes which may be downstream targets for AMKL treatment.

## Materials and Methods

### Clinical Samples

Diagnostic AML blasts (including blasts with the AMKL phenotype) were obtained from the Children’s Hospital of Michigan leukemia cell bank and from the Pediatric Oncology Group 9421 study, as previously described [18]. The diagnosis of AMKL was confirmed by flow cytometry detection of the megakaryocytic antigens CD41 and CD61. Mononuclear cells were isolated on Ficoll-Hypaque gradients to obtain highly purified mononuclear cell fractions consisting mostly of leukemic blasts. Written informed consent was provided by the parent or legal guardian of the patient according to the Declaration of Helsinki. The research protocol was approved by the Human Investigation Committee of Wayne State University School of Medicine.

### Cell Culture and Chemotherapy Agents

The Meg-01 megakaryocytic cell line was obtained from the American Type Culture Collection (Manassas, VA). The parental and engineered sublines were cultured in RPMI 1640 with 10% fetal bovine serum (FBS) (Hyclone, Logan, UT) and 2 mM L-glutamine plus 100 U/ml penicillin and 100 µg/ml streptomycin, in a 37°C humidified atmosphere containing 5% CO_2_/95% air. Ara-C, DNR and VPA were purchased from Sigma-Aldrich (St. Louis, MO).

### shRNA Knockdown of GATA1 in Meg-01 Cells

GATA1 shRNA lentivirus clones were purchased from the RNAi Consortium of Sigma-Aldrich. Meg-01 cells were transduced with the GATA1 shRNA lentivirus. After selection with puromycin, infected Meg-01 cells were plated in soft agar. Colonies were isolated, expanded and assessed for GATA1 expression by Western blotting and real-time RT-PCR. Two clones with decreased GATA1 expression (designated GATA1 4-14 and GATA1 5-13) were chosen for further study. A pool of cells from the negative control transduction (lentivirus expressing a shRNA with limited homology to any known human genes) was used as the control (designated GATA1 Neg).

### Quantitation of Gene Expression by Real-time RT-PCR

Transcripts were quantitated using primers (Table S1) and Sybr Green (Roche Diagnostics, Indianapolis, IN) as previously described [19] or Taqman probes (Applied Biosystems Inc., Foster City, CA) and a LightCycler real-time PCR machine (Roche Diagnostics, Indianapolis, IN) based on the manufacturer’s instructions. Real-time PCR experiments were expressed as mean values from three independent experiments and normalized to GAPDH, with the exception of GATA1 transcript levels post VPA treatment, which were normalized to RPL13A levels as RPL13A has been reported to be a more reliable housekeeping gene post-HDAC inhibitor treatment [20].

### Western Blot Analysis

Whole cell lysates were prepared by sonication in hypotonic buffer (10 mM Tris-Cl, pH 7.0), containing 1% SDS and proteolytic inhibitors, and subjected to SDS-PAGE. Separated proteins were electrophoretically transferred to PVDF membranes (Thermo Fisher Inc., Rockford, IL) and immunoblotted with antibodies to GATA1 (C-20, Santa Cruz Biotechnology, Santa Cruz, CA), Bcl-xL (Cell Signaling Technology, Danvers, MA), or β-actin (Sigma-Aldrich, St. Louis, MO) as described previously [19]. Immunoreactive proteins were visualized using the Odyssey Infrared Imaging System (Li-Cor, Lincoln, NE), as described by the manufacturer.

### In Vitro Ara-C and DNR Cytotoxicity Assays

For determinations of cytotoxicities, the cell lines were cultured in complete medium with dialyzed fetal bovine serum in 96-well plates at a density of 4 x 10^4^ cells/ml for 96 hours. Cells were cultured continuously with a range of ara-C and DNR concentrations at 37 °C, and viable cells were determined using the Cell Titer-blue reagent (Promega, Madison, WI) and a fluorescence microplate reader. The IC_50_ values were calculated as the concentrations of drug necessary to inhibit 50% growth compared to control cells cultured in the absence of drug. The data are presented as the mean values ± standard errors from at least 3 independent experiments. Standard isobologram analysis was performed as described previously [21] and combination index (CI) analysis was performed using CompuSyn software (ComboSyn, Inc., Paramus, NJ)

### Assessment of Baseline and Drug Induced Apoptosis

The Meg-01 shRNA stable clones (GATA1 Neg, GATA1 4-14, and GATA1 5-13) in logarithmic growth phase in RPMI 1640/10% dialyzed FBS in the presence or absence of ara-C and VPA were harvested, vigorously pipetted and triplicate samples taken to determine baseline and drug-induced apoptosis using the Annexin V-FITC Kit (Beckman Coulter, Brea, CA), as previously described [21,22]. Apoptotic events were recorded as a combination of Annexin V+/PI- (early apoptotic) and Annexin V+/PI+ (late apoptotic/dead) events. The data are presented as mean percentages of Annexin V positive cells ± standard errors relative to untreated cells.

### Chromatin Immunoprecipitation (ChIP) Assay

ChIP assays were performed in Meg-01 cells as previously described [23], with GATA1 C-terminus (C-20 antibody, Santa Cruz) antibody or normal IgG. Standard PCR for the Bcl-xL promoter region was performed with forward (5’-gcatccccgcagccacctcctc-3’) and reverse (5’-ccctaaaaattccattccccctccag-3’) primers spanning positions -257 to +67. A separate region (exon 3) of the human *GATA1* gene was also amplified with forward (5’ tggagactttgaagacagagcggctgag-3’) and reverse (5’-gaagcttgggagaggaataggctgctga-3’) primers to validate the specificity of the ChIP assays.

### ChIP-on-Chip Assay

The ChIP-on-ChIP protocol was modified from the Agilent Technologies Mammalian ChIP-on-ChIP Kit protocol. Briefly, genomic DNA from the ChIP assay above was incubated with T4 DNA polymerase to create blunt ends. Linker DNA was ligated to the blunt end DNA, followed by amplification of the samples. The samples were labeled, hybridized to the microarray, washed and scanned according to the manufacturer’s protocol. Data were imported into Chip Analytics software (v 1.3.1, Agilent Technologies) for analysis. Normalization was performed using intra-array lowest and inter-array median normalizations. Peak detection was performed using the Whitehead error model (v 1.0) and the Peak Shape Detection algorithm (v2.0). Genes with a bound region (peak) were identified, and the associated NCBI Refseq accession numbers were used for subsequent analyses. The ChIP-on-ChIP results were validated by both regular PCR and real-time PCR (see above). We have deposited the raw data at GEO under accession number GSE43018.

### Gene Expression Microarray Analysis

Gene expression microarray was performed with the Agilent Whole Human Genome 4 x 44K microarray (catalog #G4112F). Microarray sample preparation, hybridization, and data analysis were described previously [24]. On each microarray, a labeled GATA1 4-14 or GATA1 5-13 sample was co-hybridized with an oppositely labeled GATA Neg sample. Two arrays were completed for the GATA1 4-14/GATA1 Neg pair and the GATA1 5-13/GATA1 Neg pair, respectively, for a total of four arrays. The two microarrays used for each clone were hybridized in a “dye swap” arrangement with opposite dye orientation to minimize the dye bias effect. Statistical analyses were performed using Rosetta Resolver® [25]. We have deposited the raw data at GEO under accession number GSE42879 and we confirm all details are MIAME compliant.

### Construction of Plasmids, Transient Transfection, and Luciferase Assay

The GATA1 expression vector, pPacGATA1, and *Bcl-x* promoter construct, pGL3B-Bcl-x-pro, were prepared as previously described [26,27]. *D*. Mel-2 cells (Invitrogen, Carlsbad, CA) were co-transfected with 1 µg of the *Bcl-x* promoter construct and 125 to 500 ng of pPacGATA1 using Fugene 6 reagent (Roche, Indianapolis, IN) as described previously [26,27]. Luciferase activities were assayed using the Single Luciferase Assay System (Promega) and normalized to total cell protein, measured by the Bio-Rad DC-protein assay kit (Bio-Rad, Hercules, CA).

### Statistical Analysis

Differences in transcript levels between distinct AML patient groups were compared using the nonparametric Mann-Whitney two-sample U test. The nonparametric Spearman rank correlation coefficient was used to analyze the relationship between GATA1 and Bcl-xL transcript levels. Statistical analyses were performed with GraphPad Prism 4.0.

## Results

### Overexpression of GATA1 in AMKL blasts is associated with chemotherapy resistance

**Figure 1:**
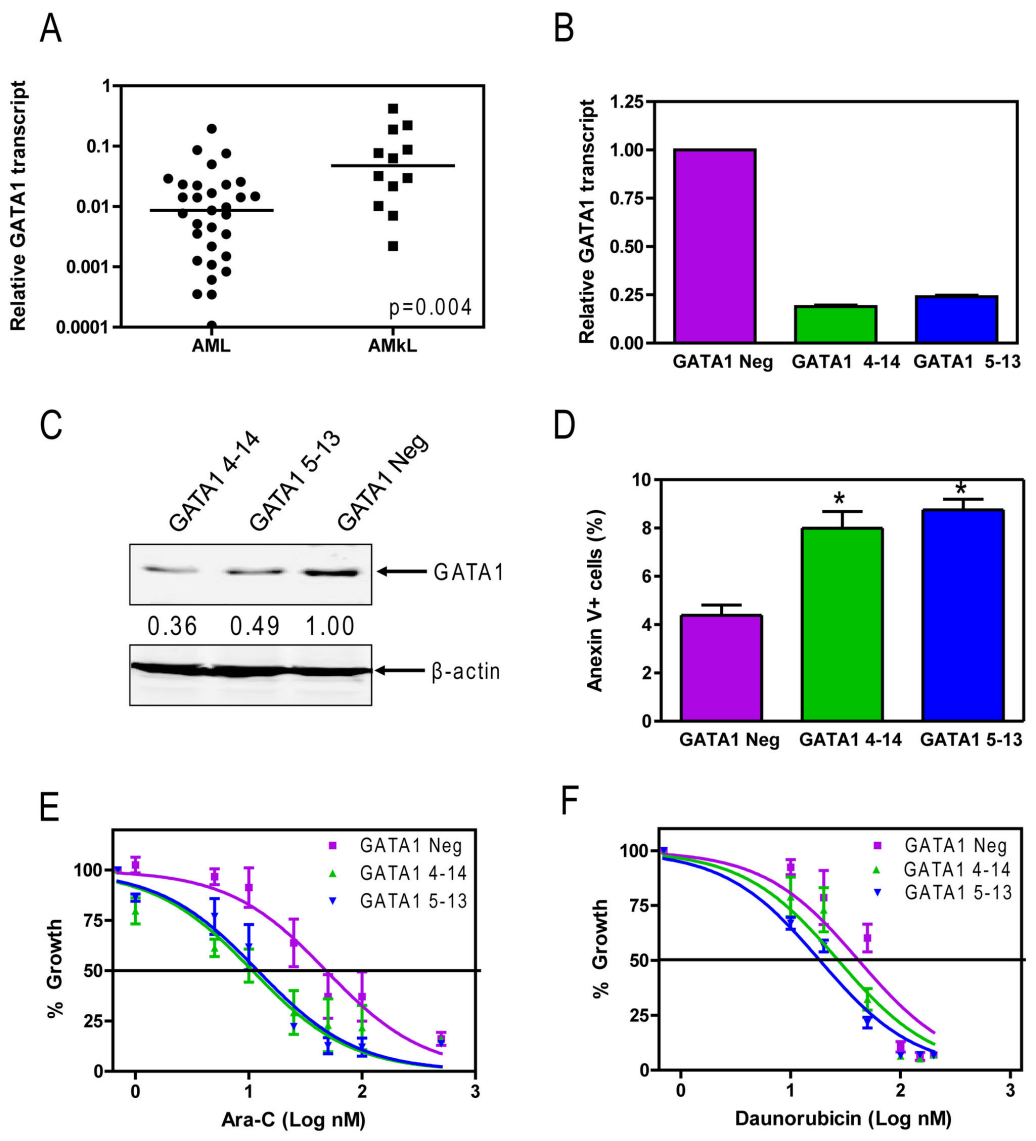
GATA1 transcripts are elevated in AMKL blasts and shRNA knockdown increases basal apoptosis and chemotherapy sensitivity. **A**: *GATA1* transcript levels in primary non-AMKL AML or AMKL blasts from non-DS patients were quantitated by real-time RT-PCR and normalized to GAPDH transcript levels. Median transcript levels were compared between the two patient groups using the nonparametric Mann–Whitney *U* test. **B**–**C**: Meg-01 cells were infected by GATA1 shRNA lentivirus clones. Colonies were isolated, expanded and tested for GATA1 expression by real-time RT-PCR (panel B) and Western blotting (panel C). Two colonies (GATA1 4-14 and GATA1 5-13) with decreased GATA1 gene expression were selected for further study. A pool of cells from the negative control infection was used as the control (designated GATA1 eg). **D**: Baseline apoptosis in the GATA1 4-14, GATA1 5-13 and GATA1 Neg was determined by flow cytometry with Annexin V-FITC/PI staining. *indicates p<0.05, error bars indicate standard errors. **E**–**F**: Meg-01 GATA1 shRNA clones were cultured in 96-well plates at a density of 4x10^4^ cells/mL in the presence of 0-500 nM Ara-C (panel C) or 0-200 nM DNR (panel D) and cell numbers were determined with the Cell Titer-blue reagent and a fluorescence microplate reader. The data represent mean values ± standard errors from at least three independent experiments.

To determine if GATA1 is overexpressed in AMKL compared to other subtypes of AML in non-DS children, real-time RT-PCR was performed to quantify transcript levels of GATA1 in a cohort of diagnostic AML blast samples (12 AMKL cases and 31 non-AMKL AML cases). GATA1 transcript levels were significantly higher in AMKL blasts compared to blasts of other AML subtypes amongst non-DS patients (median 5.5-fold, p=0.004) (Figure 1A).

To explore the role of GATA1 in chemotherapy sensitivity in AMKL, GATA1 expression was knocked-down using lentivirus shRNA in the non-DS megakaryocytic cell line Meg-01. Two stable clones, designated GATA1 4-14 and GATA1 5-13, showed decreased *GATA1* transcripts [~25% relative to a non-targeted control comprised of a pool of cells infected with a lentivirus negative control shRNA (designated GATA1 Neg)], while at the protein level, GATA1 was reduced to approximately one-third and one-half that of GATA1 Neg in the GATA 4-14 and GATA 5-13 clones, respectively (Figure 1B& C). This was accompanied by significantly increased baseline apoptosis in both clones relative to the GATA1 Neg cells, as measured by flow cytometry with Annexin V-FITC/PI staining (Figure 1D). Down-regulation of GATA1 also resulted in increased anti-proliferative activity for ara-C and DNR, as measured by the Cell Titer-Blue viability assay. The IC_50_s for ara-C in the GATA1 Neg, GATA1 4-14, and GATA1 5-13 were 48.3 nM, 10.8 nM, and 12.0 nM, respectively, while those for DNR were 41.6 nM, 27.1 nM, and 18.4 nM, respectively (Figure 1E& F). This sensitivity was reduced, at least in part, by exogenous expression of Bcl-xL in the GATA1 4-14 clone (Figure S1B& C)

### Bcl-xL is overexpressed in AMKL and is a GATA1 target gene

**Figure 2:**
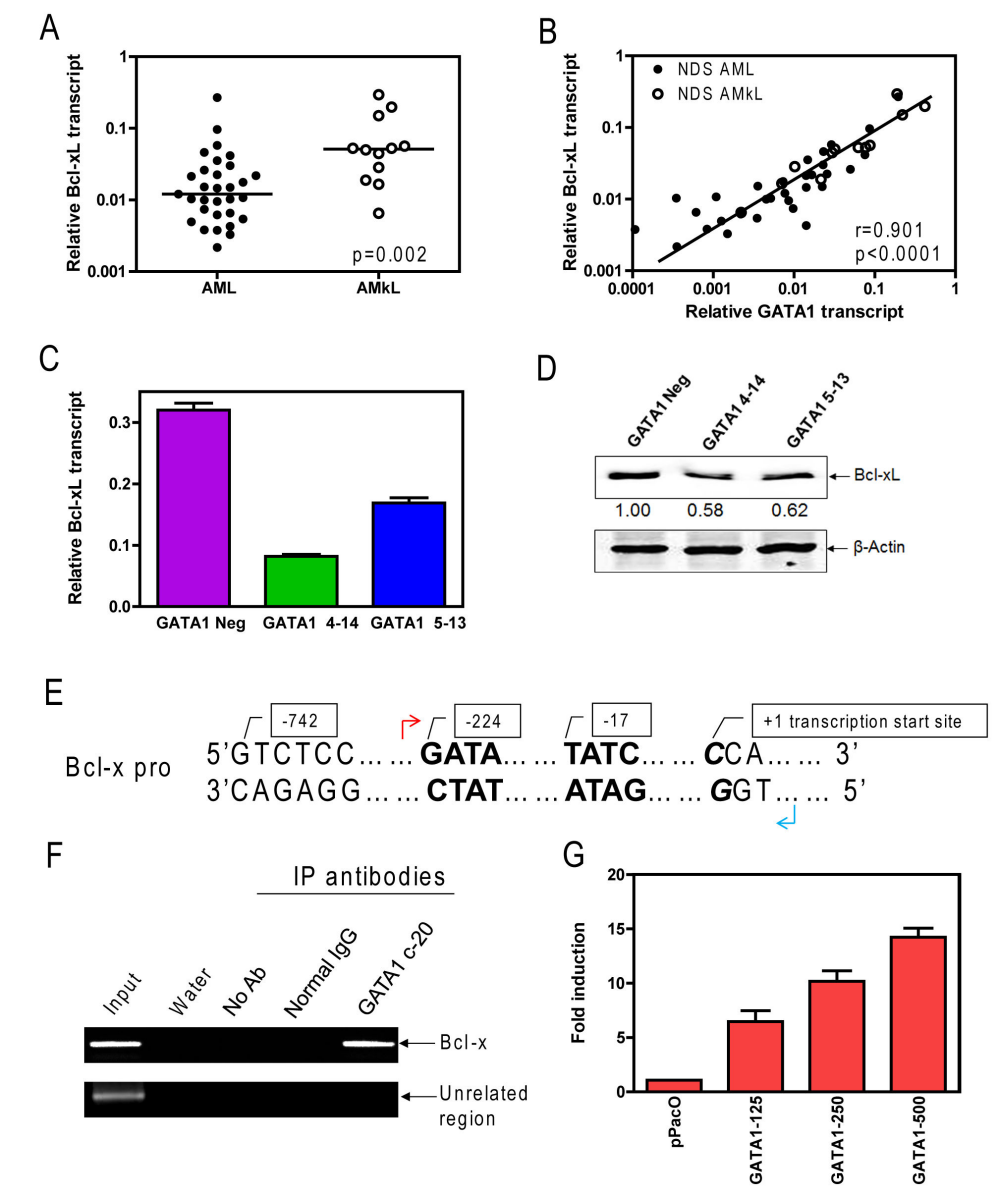
Bcl-xL is a *bona fide* GATA1 target gene in AMKL. **A**: *Bcl-xL* transcript levels in primary non-AMKL AML or AMKL blasts from non-DS patients were quantified by real-time RT-PCR. Median transcript levels were compared between the two patient groups with the use of the nonparametric Mann–Whitney *U* test. **B**: The relationship between *GATA1* and *Bcl-xL* transcript levels was determined by the nonparametric Spearman rank correlation coefficient. **C**: Transcript levels for *Bcl-xL* were quantified by real-time RT-PCR in the Meg-01 shRNA stable clones. Real-time PCR results were expressed as mean values from three independent experiments and normalized to GAPDH. **D**: Whole cell extracts from Meg-01 GATA1 Neg, 4-14 and 5-13 were subjected to Western blotting and probed for Bcl-xL and β-actin. **E** and **F**: In vivo binding of GATA1 to the putative GATA1 binding sites located in the upstream region of the *Bcl-x* gene in Meg-01 cells was determined by ChIP assays with the use of regular PCR (panel F), as described in the “Materials and Methods”. The location of the primers used in 2F are indicated by the arrows in 2E. **G**: *D*. Mel-2 cells were transfected with pGL3Basic-Bcl-x-pro along with 0-500 ng of pPacGATA1 using the Fugene 6 reagent. GATA1 transactivation of *Bcl-x* promoter was determined by luciferase reporter gene assays and normalized to total cell protein.

It was previously reported that GATA1 promotes survival of developing erythrocytes by upregulating Bcl-xL [16]. On this basis, we hypothesized that GATA1 could play a potential role in the survival of AMKL blasts upon treatment with cytotoxic agents by upregulating BcL-xL. To begin to test this possibility, Bcl-xL transcripts were initially measured by real-time RT-PCR in the above cohort of 31 AMKL and 12 non-AMKL AML cases. Consistent with our hypothesis, Bcl-xL transcript levels were significantly higher (median 4.2-fold; p=0.002) in AMKL cases compared to that in other AML subtypes (Figure 2A). The transcript levels of Bcl-xL closely correlated with GATA1 transcripts in AML and AMKL blasts (r=0.901, p<0.0001) (Figure 2B). Further, in the cell line models, knockdown of GATA1 resulted in decreased Bcl-xL expression at both the transcript and protein levels (Figure 2C& D). Bcl-2 protein was not detected by western blot in these cells and Mcl-1 levels were comparable to the GATA-1 Neg cells (Figure S1A).

To provide evidence that GATA1 directly activates expression of Bcl-xL by binding to the putative GATA1 sites in the *Bcl-x* gene promoter region (Figure 2E), ChIP was performed in Meg-01 cells followed by PCR of the recovered DNA using primers flanking the putative GATA1 binding sites. As can be seen in Figure 2F, pull down with the GATA1-specific antibody GATA1 c-20 resulted in specific precipitation of the *Bcl-x* promoter region (containing the two putative GATA1 binding sites) but not an unrelated region. Activation of the *Bcl-x* promoter activity by GATA1 was further confirmed using a luciferase reporter assay after cotransfection of *D*. Mel-2 cells with increasing amounts of a GATA1 expression vector along with the *Bcl-x* promoter construct. As shown in Figure 2G, increasing GATA1 transfection resulted in dose-dependent induction of luciferase expression driven by the *Bcl-x* promoter. Taken together, these studies provide direct evidence that GATA1 binds to the GATA1 sites in the *Bcl-x* upstream region and transactivates its activity in Meg-01 cells.

### Treatment with VPA down-regulated GATA1 and Bcl-xL and sensitized Meg-01 cells to ara-C-induced apoptosis

**Figure 3:**
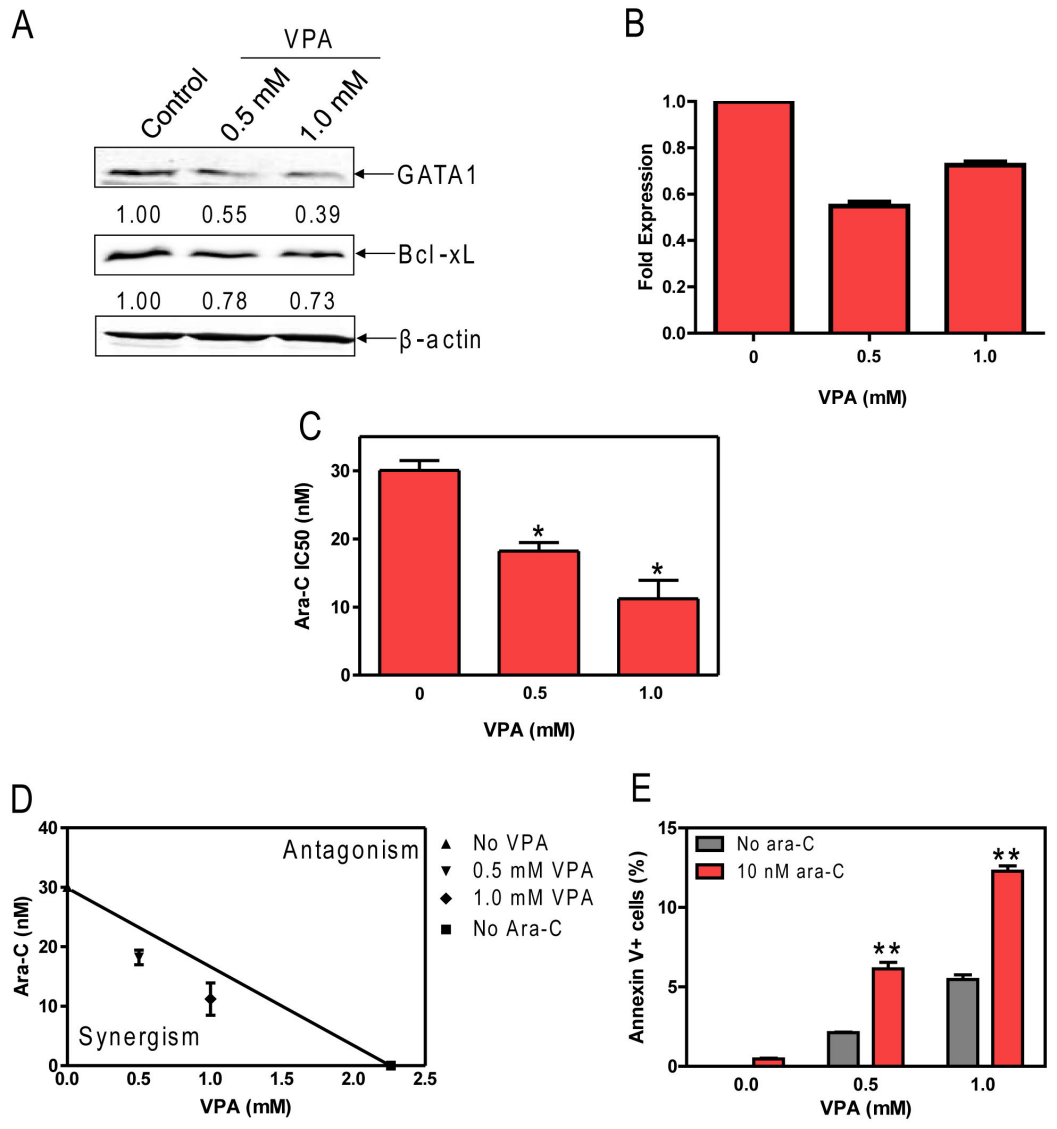
Valproic acid causes down-regulation of GATA1 and enhances ara-C induced apoptosis in Meg-01 cells. **A**: Meg-01 cells were treated with VPA for 48h. Whole cell lysates were extracted, subjected to Western blotting and probed by anti-GATA1, Bcl-xL and β-actin. **B**: Meg-01 cells were treated at the indicated dose of VPA for 48h and RNA was harvested for quantification of GATA1 transcripts by qRT-PCR. Transcript levels for GATA1 in the VPA treated cells were normalized to untreated cells. **C**: Meg-01 cells were treated with ara-C in the presence or absence of VPA and viable cell numbers were determined using Cell Titer-blue reagent. IC_50_ values were calculated as the concentration of drug necessary to inhibit 50% proliferation compared to control cells cultured in the absence of drug. * indicates p<0.05 **D**: Standard isobologram analysis of Meg-01 cell proliferation inhibition by the combined treatment of ara-C and VPA. The IC50 value for each drug is plotted on the axes; the solid line represents the additive effect, while the points represent the concentrations of each drug resulting in 50% inhibition of proliferation. Points falling below the line indicate synergism between drug combinations whereas those above the line indicate antagonism. The data are presented as mean values ± standard errors from at least 3 independent experiments. **E**: Apoptosis after indicated treatment for 48 hours as determined by flow cytometry with Annexin V-FITC/PI staining. *indicates p<0.05 relative to no ara-C, error bars indicate standard errors.

With currently available treatments, it is not feasible to directly target GATA1 in AMKL cells. Based on a previous report [28], we hypothesized that treatment with VPA, an HDAC inhibitor, could potentially down-regulate GATA1 expression. Indeed, treatment of Meg-01 cells with clinically achievable concentrations (0.5 and 1.0 mM) of VPA resulted in a reduction in GATA1 protein expression, as measured by western blot (Figure 3A), while treatment with comparably toxic doses of DNR did not (not shown). Furthermore, VPA treatment also resulted in decreased expression of Bcl-xL protein (Figure 3A). To investigate the mechanism through which VPA modulates GATA1 expression, real time RT-PCR was used to look at GATA1 transcript levels post VPA treatment. Transcript levels were decreased after both 0.5 and 1.0 mM treatments, albeit to different degrees (Figure 3B).

To determine the impact of VPA treatment on ara-C cytotoxicity, we measured the viability of Meg-01 cells upon treatment with ara-C treatment along with increasing VPA concentrations using the Cell Titer-Blue viability assay. When simultaneously administered with ara-C, VPA at 0.5 and 1 mM significantly enhanced ara-C sensitivity (as reflected in decreased IC_50_s) by 1.6- and 2.7-fold, respectively (Figure 3C). The combined effects of ara-C with VPA on cell proliferation were clearly synergistic, as determined by standard isobologram analysis (Figure 3D) and by calculating CI values. A CI<1 (0.83 and 0.81 for the combination with 0.5 and 1.0 mM VPA, respectively), indicative of synergism, was calculated for each of the drug combinations. In addition, the combination of VPA and ara-C synergistically induced apoptosis as measured by flow cytometry with Annexin V-FITC/PI staining (Figure 3E). Exogenous expression of Bcl-xL in Meg-01 cells abrogated the enhancement of ara-C cytotoxicity by VPA and caused resistance to VPA or ara-C (Figure S1B& D)

### Identification of additional GATA1 target genes

**Figure 4:**
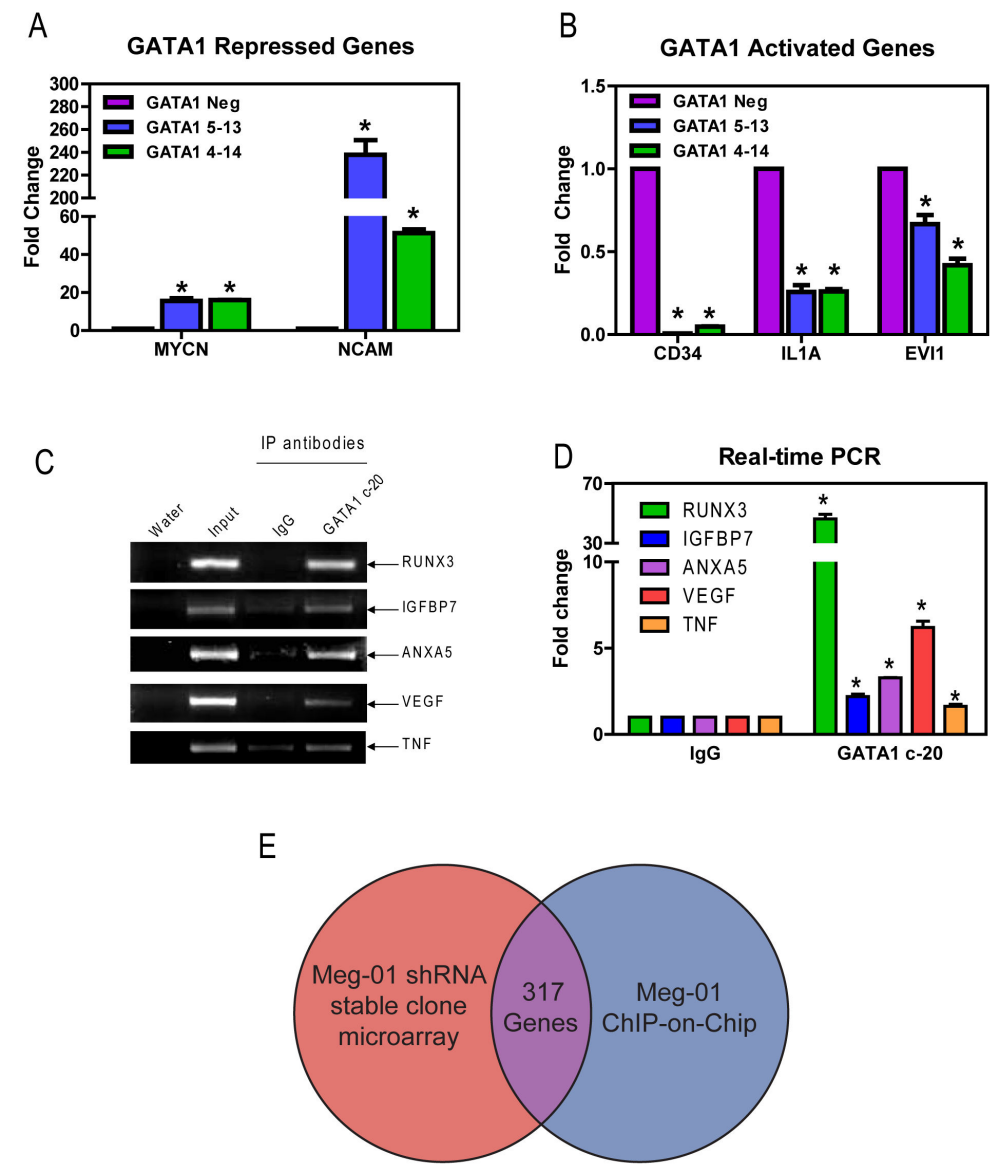
Identification of additional GATA1 target genes. **A** and **B**: Oligonucleotide microarray analysis was performed with RNA samples form the Meg-01 shRNA stable clones and the results were validated by real-time RT-PCR using Taqman probes. GATA1-repressed (panel A) and GATA1-activated (panel B) transcript levels for MYCN, NCAM1, CD34, ILA1, and EVI1 are expressed as mean values from three independent experiments and normalized to GAPDH. **C** and **D**: ChIP-on-Chip array analysis was performed in Meg-01 cells and the results were validated by PCR (panel C) and real-time PCR (panel D) using primers against each region (Table S1). **E**: Overlapping genes between oligonucleotide microarray and ChIP-on-Chip array were identified by cross-reference of the two sets of data using GenBank accession numbers.

In order to identify additional GATA1 target genes that may contribute to chemotherapy resistance, oligonucleotide microarray analyses on RNAs from the GATA1 5-13/4-14 clones and GATA1 Neg control were performed. Average log ratios, representing the differences in expression between the *GATA1* shRNA clones and the GATA1 Neg control, were derived for each array feature by combining replicate array data, using the error-weighted algorithm of Rosetta Resolver®. Differentially expressed genes were identified by their p-values, calculated with the Resolver error-model and the replicate data. Using a p-value ≤ 0.005 as cutoff, 3210 differentially expressed features (probes) were identified, with a false discovery rate of 6.4%. Of these, 1521 were down regulated in the knockdown clones, while 1689 were upregulated. Validation of the microarray results was performed using real-time RT-PCR and separate RNA samples from the Meg-01 stable clones. As shown in Figure 4A-B, real-time RT-PCR was able to validate both up- and down-regulated genes. A full list of differentially expressed probes can be found in Tables S2 and S3. Interestingly, the down-regulated genes were significantly enriched for genes involved in regulating cell division and cell death, and a large number of the upregulated genes were associated with chromatin assembly and organization (Tables S4 and S5, respectively), as determined by DAVID gene functional annotation analysis [29,30].

The above oligonucleotide microarray experiment detected both direct and indirect GATA1 target genes in Meg-01 cells. To identify direct targets of GATA1 among the oligonucleotide microarray gene list, a ChIP-on-Chip experiment was performed. Validation of the ChIP-on-Chip analyses was performed using both regular PCR and real-time RT-PCR (Figure 4C-D). The gene accession numbers associated with ChIP-on-Chip peaks (described in methods) were compared to the accession numbers for the 3210 differentially expressed genes in the GATA1 knockdown clones. The cross-referencing produced a list of 317 common genes (Figure 4E Table S6) which is likely to be highly enriched for *bona fide* GATA1 target genes. Interestingly, there were many genes in this overlapping group that were found by DAVID ontology analysis to be associated with either regulation of cell death, cell cycle, or proliferation (Table 1.).

**Table 1:** Overlapping genes associated with cell-cycle, apoptosis, or proliferation.

Accession Code	Name	Fold Change	P-value
NM_006744	RBP4	-3.116	8.89E-16
NM_002253	KDR	-3.081	1.14E-41
NM_000921	PDE3A	-2.489	7.30E-09
NM_030751	TCF8	-2.144	3.70E-03
NM_002371	MAL	-1.911	2.68E-15
NM_021120	DLG3	-1.749	1.02E-07
NM_000459	TEK	-1.464	1.42E-15
NM_001005333	MAGED1	-1.403	5.35E-18
NM_003879	CFLAR	-1.336	1.33E-06
NM_000061	BTK	-1.290	2.70E-03
NM_003292	TPR	-1.274	3.94E-06
NM_001204	BMPR2	-1.242	2.39E-11
NM_004360	CDH1	-1.216	1.50E-03
NM_001154	ANXA5	-1.195	9.00E-04
NM_001006	RPS3A	-1.174	4.08E-07
NM_001605	AARS	-1.125	2.30E-03
NM_138957	MAPK1	1.094	2.60E-03
NM_003318	TTK	1.120	1.20E-03
NM_016542	MST4	1.130	4.50E-03
NM_013239	PPP2R3B	1.146	1.00E-04
NM_003246	THBS1	1.151	4.00E-04
NM_001813	CENPE	1.151	3.50E-03
NM_014881	DCLRE1A	1.156	2.20E-03
NM_006729	DIAPH2	1.173	3.15E-06
NM_018451	CENPJ	1.187	1.00E-04
NM_000876	IGF2R	1.225	4.60E-03
NM_002710	PPP1CC	1.226	4.00E-04
NM_033031	CCNB3	1.236	1.00E-04
NM_032375	AKT1S1	1.247	3.47E-05
NM_000675	ADORA2A	1.247	4.00E-03
NM_019619	PARD3	1.266	1.50E-03
NM_138375	CABLES1	1.277	2.00E-04
NM_002291	LAMB1	1.285	3.59E-07
NM_001104	ACTN3	1.289	1.70E-03
NM_001003940	BMF	1.380	3.43E-05
NM_000599	IGFBP5	1.480	2.80E-03
NM_000600	IL6	1.604	7.75E-08
NM_003239	TGFB3	1.615	3.76E-05
NM_006006	ZBTB16	1.615	1.88E-07
NM_053056	CCND1	1.654	2.84E-08
NM_000852	GSTP1	1.802	7.00E-04
NM_006147	IRF6	1.917	7.00E-04
NM_000417	IL2RΑ	2.419	3.15E-08
NM_003991	EDNRB	2.662	5.31E-12
NM_005378	MYCN	4.948	2.10E-03

## Discussion

Despite progress in the treatment of AML, there are still AML subtypes such as AMKL that have a poor prognosis. Hence, studies examining the basis for the relative chemotherapy resistance of these subgroups may lead to improvements in therapy. The significantly higher cure rates of DS AMKL patients, who almost uniformly harbor somatic mutations in the *GATA1* gene, suggested that GATA1 may play a critical role in chemotherapy response and resistance [3,5–9]. This is also supported by studies which established an association between high expression levels of GATA1 and a poorer prognosis in adult AML [12,13]. This is particularly relevant to non-DS AMKL since overexpression of GATA1 in this AML subtype has been previously observed [11]. However, the molecular mechanism by which GATA1 confers chemotherapy resistance in AMKL remains unknown.

This study established that for patients whose leukemic blasts express high levels of GATA1, one potential mechanism of resistance involves overexpression of the anti-apoptotic protein Bcl-xL. A direct role for GATA1 in the expression of Bcl-xL was implicated by knocking down GATA1 in the megakaryocytic cell line, Meg-01. Upon knocking down GATA1, Bcl-xL expression was partially abrogated, accompanied by increased basal apoptosis and sensitivity to ara-C and DNR. Although this finding was not entirely unexpected, as the Bcl-2 family proteins are known to inhibit apoptosis and lead to chemotherapy resistance, and Bcl-xL has a well-documented role in the maintenance of the megakaryocyte lineage [31], it nonetheless suggests a potential target for patients with AMKL. The Bcl-2 family inhibitor GX15-070 is currently undergoing clinical trials (www.clinicaltrials.gov) and has shown single agent efficacy in leukemia patients [32,33].

Another potential approach to enhance the treatment of AML with high expression of GATA1 involves the use of HDAC inhibitors combined with standard chemotherapy regimens. While HDAC inhibitors are well known to exhibit modest anti-leukemic activity as single agents, preclinical work from our group has demonstrated their strong enhancement of standard agents [21,34]. In this study, we showed that VPA, an FDA approved anti-epileptic medicine that also acts as an HDAC inhibitor, was able to down-regulate GATA1 along with Bcl-xL, resulting in synergistic induction of apoptosis upon addition of ara-C. Changes in transcript levels post-VPA treatment did not account for the total differences in GATA1 protein seen in this study (Figure 3A-B), suggesting that VPA affects both the transcriptional and post-transcriptional regulation of GATA1. Current clinical trials are investigating the role of HDAC inhibitors as adjuvant therapy in many cancers, including pediatric AML (www.clinicaltrials.gov).

While Bcl-xL appears to be an important GATA1 target, it was of interest to identify additional genes that were regulated either directly or indirectly by GATA1. Using gene expression microarray analysis, we were able to identify over 3000 genes that were differentially expressed upon GATA1 knockdown including genes involved in regulating chromatin structure and cell death. By cross-referencing the differentially expressed genes with regions identified by ChIP-on-Chip, we were able to generate a list of 317 genes corresponding to *bona fide* GATA1 targets. In addition to those, other potentially important genes were identified. One such gene is DYRK1A, which encodes the dual-specificity tyrosine-(Y)-phosphorylation regulated kinase 1A. This gene was recently identified as a driver of megakaryocytic leukemia in a mouse DS AMKL model [35]. Neural Cell Adhesion Molecule (NCAM), which was found to be repressed by GATA1, is associated with poorer prognosis in AML [36] and early death in pediatric AML patients [37]. Vascular endothelial growth factor (VEGF) was identified by the ChIP-on-ChIP to be a direct GATA1 target is indicative of poorer prognosis [38] and is also a potential therapeutic target in AML [39]. Both Evi1 and CD34 were found to be activated by GATA1 and could potentially contribute to the poorer outcome found in GATA1 overexpressing patients. Evi1 has been found to be epigenetically deregulated and associated with poor prognosis in AML [40]. CD34 is a well-established surface marker present on immature hematopoietic cells that despite having variable prognostic capacity in mixed AML backgrounds [41], is of prognostic significance within some subgroups [42,43]. Though not specifically investigated here, this may suggest a potential role for the reduction of GATA1 levels in the differentiation induced by HDAC inhibitors in AML cells [44].

In summary, in this study we were able to further clarify the role of GATA1 in AMKL. Blasts from patients with AMKL overexpressed GATA1 relative to those with other AML subtypes. The finding that GATA1 is able to bind and activate the *Bcl-x* promoter coupled with the high correlation between GATA1 expression and Bcl-xL transcript levels in primary patient samples offers a potential explanation for why AML patients with high GATA1 expression have been found to have poorer outcomes. Furthermore, treatment with the HDAC inhibitor, VPA, was shown to decrease both GATA1 and Bcl-xL expression in Meg-01 cells and sensitize them to treatment with ara-C. Finally, by combining gene expression microarray and ChIP-on-Chip analyses, we were able to identify additional GATA1 target genes to serve as a basis for future studies of both potential chemotherapeutic interventions and AMKL biology

## Supporting Information

Figure S1 Effect of GATA-1 knockdown on Bcl-2 and Mcl-1 expression and overexpression of Bcl-xL overcomes ara-C sensitivity resulting from GATA1 knockdown and conveys resistance to VPA. A Western blots demonstrating the impact of GATA-1 knockdown on Bcl-2 and Mcl-1 expression. 100µg of protein were loaded in each lane, with an excess of THP-1 lysate as a positive control (far right). B Western blots demonstrating overexpression of Bcl-xL in both the parental Meg-01 and Meg-01 4.14 cell lines. B–C Indicated cells were treated for 24 hours at the indicated drug dose and viability was determined using trypan blue exclusion. * indicates p < 0.05 compared to RFP or between indicated columns.
(PDF)

Table S1 Summary of primers used for PCR validation of GATA1 ChIP on ChIP. (XLSX)

Table S2 Probes with lower expression after GATA1 knockdown in Meg-01 cells. (XLSX)

Table S3 Probes with higher expression after GATA1 knockdown in Meg-01 cells. (XLSX)

Table S4 Genes associated with cell division and cell death. (XLSX)

Table S5 Genes associated with chromatin assembly and organization. (XLSX)

Table S6 Overlapping genes between ChIP-on-Chip and gene expression microarray. (XLSX)

Methods S1 (DOCX)
