# Supplementary figures and images for: Overexpression of GATA1 Confers Resistance to Chemotherapy in Acute Megakaryocytic Leukemia
Source: PLoS One. 2013 Jul 10;8(7):e68601. doi: 10.1371/journal.pone.0068601 (PMC3707876; doi:10.1371/journal.pone.0068601)

Figure S1

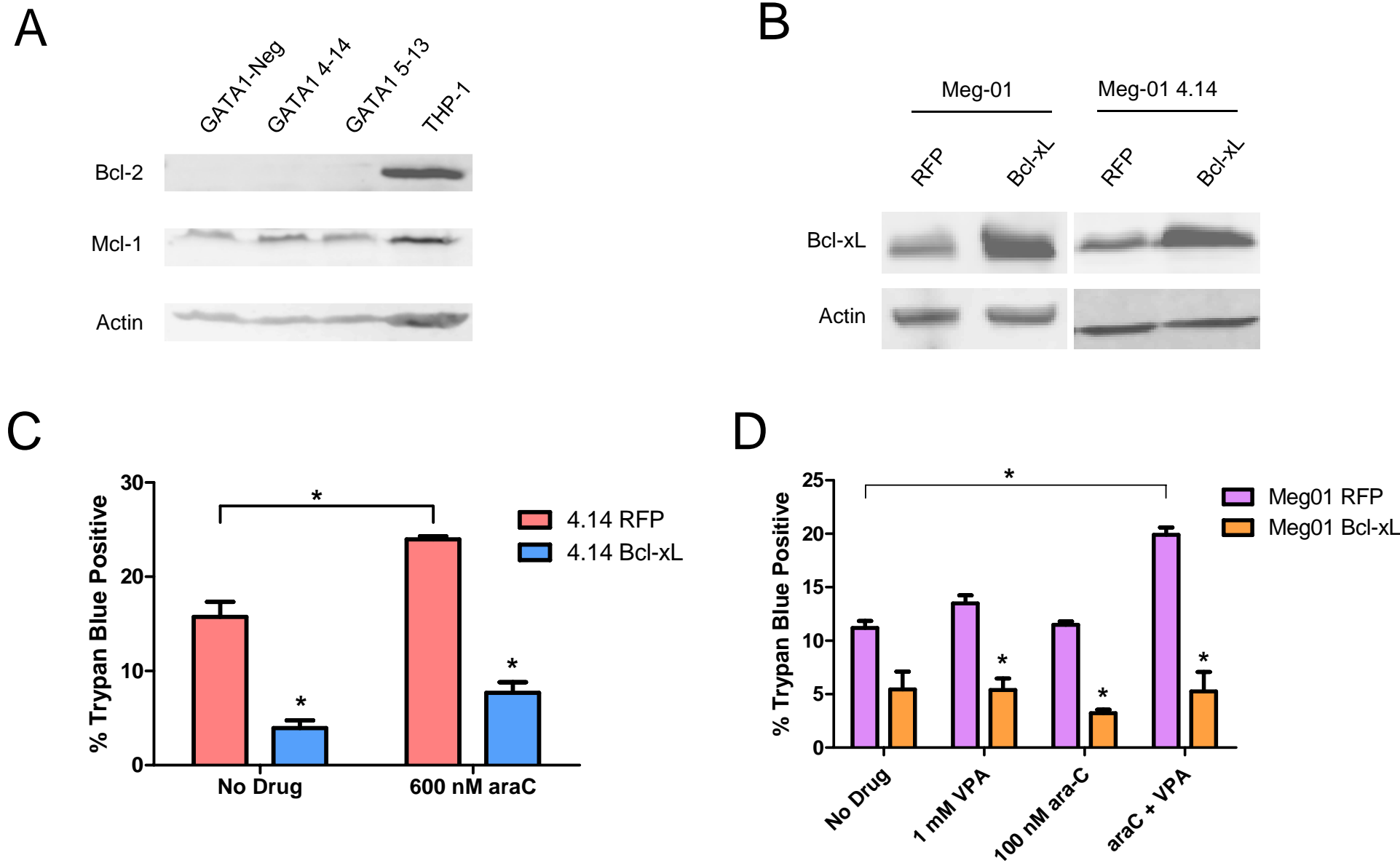

Supplement: Figure S1 — A Western blots demonstrating the impact of GATA-1 knockdown on Bcl-2 and Mcl-1 expression. 100µg of protein were loaded in each lane, with an excess of THP-1 lysate as a positive control (far right). B Western blots demonstrating overexpression of Bcl-xL in both the parental Meg-01 and Meg-01 4.14 cell lines. B–C Indicated cells were treated for 24 hours at the indicated drug dose and viability was determined using trypan blue exclusion. * indicates p < 0.05 compared to RFP or between indicated columns. (PDF) [file pone.0068601.s001.pdf]
