# Supplementary material for: Overexpression of GATA1 Confers Resistance to Chemotherapy in Acute Megakaryocytic Leukemia
Source: PLoS One. 2013 Jul 10;8(7):e68601. doi: 10.1371/journal.pone.0068601 (PMC3707876; doi:10.1371/journal.pone.0068601)
Supplement: Methods S1 — (DOCX) [file pone.0068601.s008.docx]

**Supplemental Methods**

***Production of Lentivirus Particles and Transduction***

The pMD-VSV-G and delta 8.2 plasmids were gifts from Dr. Dong at the Tulane University. The transfection was carried out by using Lipofectamine and Plus reagents (Life Technologies, Carlsbad, CA) according to the manufacturer’s instructions. Briefly, of RFP (red fluorescent protein) or Bcl-xL cDNA construct from Thermo Fisher Scientific Biosciences (Lafayette, CO), pMD-VSV-G and delta 8.2 were cotransfected into TLA-HEK293T cells and the culture medium was harvested 48 h post-transfection. 2x10^5^ cells were transduced overnight by adding 1 ml of virus supernatant and 4 µg of polybrene (Sigma-Aldrich). Cells were then triple washed and allowed to grow for 24-48 hours before addition of blasticidin for selection. Cells were selected for at least 7 days before being used for experiments. (Invivogen, San Diego, CA).

***Trypan Blue Exclusion Assay***

An aliquot of the indicated cells was mixed 1:1 with a 0.4% trypan blue solution (Sigma-Aldrich, St. Louis, MO) and allowed to incubate for 30 seconds. The mixture was loaded into a haemocytometer and total cell counts as well as trypan blue positive cell counts were made. Reported results are the percentage of the total cells that were trypan blue positive and represent the average of at least three independent counts.
